# Thot: An Exalted Surgeon and Physician

**Published:** 1898-06

**Authors:** Frank Bushnell


### THOT: AN EXALTED SURGEON AND PHYSICIAN.

BY

FRANK BUSHNELL, M.D., B.S. Lond., M.R.C.S. Eng.

---

PIETSCHMANN[1] has collected very interesting evidence from classical writers, Egyptian papyri, and monuments, of the god Thot, as a surgeon and physician. Although legendary, it is probably the earliest reference to the science and art of medicine, and is the embodiment of the earliest human ideas known to us on the subject.

Thot, the feudal divinity of Hermopolis in Middle Egypt, was represented by the sacred ibis and the cynocephalus. From earliest times he possessed a harem, in which however were only two wives—Nahmâûît, "she who removes evil," and Seshaît. Every traveller up the Nile has seen figures of them carved on the temple of Denderah. Like all other gods Thot possessed a soul and body, and through his body flowed the mysterious fluid "Sa," which carried health, vigour, and life, and he could communicate this to human beings by placing his hand on the nape of the patient's neck. By his incantations he could control

[1] *Hermes Trismegistos*, p. 20 et seq., 43 et seq., 57.

the progress of the Sun-bark; and he was himself a lunar god, who measured time, counted the days, numbered the months, and commanded the forces of the universe. He was Lord of the voice, Master of words and books, inventor of those magic writings which nothing in Heaven, or Earth, or Hades could withstand.[1]

In two pottery figures he is represented as a sacred ibis, which holds the goddess of Truth suspended from its beak by a ring; and also as a baboon or cynocephalus, who sits on his haunches with his forepaws on his knees, and in his eyes is a look of grave yet impenetrable wisdom.

On one occasion he played draughts with the moon, and won a seventy-second of its fires. With this he made five days and added them to the year, and so enabled Nûit to bring forth her five children. Râ had cast a spell over Nûit to prevent this happening on any month of any year, and so Thot managed to circumvent the Sun god. He also accompanied Isis on her melancholy journey to collect the fourteen pieces of the body of her murdered husband, Osiris. By Thot's help Isis succeeded, with the exception of one piece, which "member" the oxyrhyncus rising from the Nile greedily devoured.

By magic Thot ameliorated the discomfort entailed by the embalment of Osiris. With Horus and Isis he inscribed the bandages with protective figures and formulæ, decorated the body with amulets, and drew scenes of life beyond the tomb,[2] and by various rites he enabled Osiris to live again.[3] On his wings of an ibis he was able to carry the soul of the dead over the Lake of Kha to the Islands of the Blest and the shores of Paradise. There he weighed the heart of the deceased in the scales of Truth, and always merciful he bore down on the side of Truth, that judgment might be lenient.[4]

From being a deity he became a divine king, and, in addition to medicine, *all* the arts and sciences emanated from him, including astronomy and magic.[5] He carried "the painted Eye of

[1] Cf. "The Tale of Satni," *Contes populaires de l'Ancienne Égypte*—Maspero.

[2] Mariette. [3] E. Schiaparelli, *Il Libro dei Funerali degli antichi Egiziani.*

[4] Naville, *Book of the Dead*, vol. i, pls. cxxxiii.–cxxxix. Maspero, *Catalogue du Musée Égyptien de Marseille.*

[5] Jablonski, *Pantheon Ægyptiorum*, vol. iii., p. 159.

the Sun."[1] He instituted a calendar, and according to it, he who entered the world on 4th, 5th or 6th of Paophi died of marsh fever, of love, or of drunkenness,[2] but with the evil he gave the remedy: a little blood, a few nail parings, or some hair might be an irresistible charm. He could combat the evil spirits which possessed human beings, and left their traces on the brain, the heart, the lungs, and intestines.

Let us turn from the physician to the diseases he professed to cure. Migraine and violent headache were attributed to dead men or women, who entered into the living.[3] The Ebers papyrus,[4] the Berlin medical papyrus,[5] and Herodotus,[6] describe the diseases of the country, many of which are now in existence, and their diagnosis and treatment with much accuracy; and also the division of the profession into general practitioners and specialists, and their (imaginary) description of physiological processes and anatomical points.

Ophthalmia, abdominal diseases, affections of the bladder, worms, varicose veins, ulcers of the leg, the Nile pimple (which I can personally vouch for exists at the present date), epilepsy (Brugsch), and anæmia (one quarter of modern Egyptians are afflicted with anæmia, chiefly from anchylostomum duodenale, and also from bilharzia hæmatobia); all existed. Gastric catarrh is thus described: "The abdomen is heavy, the pit of the stomach painful, the heart burns and palpitates violently. The clothing oppresses the man and he can barely support it. Nocturnal thirsts. His heart is sick, as that of a man who has eaten of the sycamore gum. . . . If he seek to supply a want of nature he finds no relief. Say to this, 'There is an accumulation of humours in the abdomen, which makes the heart sick. I will act.'"[7]

The remedies were nearly anything that could be swallowed, solid or liquid. (Chabas, Brugsch, Stern, and Lüring.) Of vege-

1 E. de Bergmann, *Historische Inschriften*, pl. lii.

2 *Sallier Papyrus IV.*, pl. iv. l. 3, pp. 4–6.

3 "The Leyden Papyrus," by Pleyte, *Études Égyptologiques*, vol. i,

4 Pl. xxxvi. l. 4, pl. xliv. l. 12, pl. xcix. l. 1—c. l. 14.

5 Pl. xv. l. 5, pl. xvi. l. 3.

6 ii. 77, 84.

7 *Medical Papyrus of Berlin*, pl. xiii. ll. 3–6.

tables, everything from herbs to trees; sawdust of the cedar was known as an antiseptic; sea salt, alum, nitre, sulphate of copper, flesh and viscera, secretions and excretions, blood and gall, "the milk of a woman who has given birth to a boy," the dung of a lion, a tortoise's brains, an old book (a book was a papyrus) boiled in oil (applied to the stomach to relieve the constipation of an infant, in other words a linseed meal poultice). The Egyptian physician was his own dispenser: fat was the vehicle for ointments, and water for draughts; but he did not despise the urine of men and animals, "the whole, sweetened with honey, was taken hot, night and morning."

Although my readers will see that the original physician was of divine origin, and enjoyed powers denied, alas, to his 19th century successors, yet we may at least lay claim to as long and as efficacious a list of remedies "to be swallowed," which list, judging from daily advertisements, is growing apace.

